# Psychopharmacological factor in the course of COVID-19 among psychiatric inpatients

**DOI:** 10.1192/j.eurpsy.2024.1050

**Published:** 2024-08-27

**Authors:** M. Sorokin, A. Shabelnik, M. Bocharova, N. Lutova, O. Limankin, L. Azarova, N. Neznanov

**Affiliations:** ^1^V.M.Bekhterev National Medical Research Centre for Psychiatry and Neurology, St. Petersburg, Russian Federation; ^2^King’s College London, London, United Kingdom; ^3^ St. Petersburg Psychiatric Hospital No. 1 named after P.P. Kashchenko; ^4^ North-Western State Medical University named after I.I. Mechnikov; ^5^FSBEI HE I.P. Pavlov SPbSMU MOH Russia, St. Petersburg, Russian Federation

## Abstract

**Introduction:**

It is known that many psychopharmacological drugs have anti-inflammatory, as well as antibacterial and antiviral effects.

**Objectives:**

To investigate the association between the severity and duration criteria of COVID-19 with psychopharmacotherapy in double-diagnosed patients.

**Methods:**

A total of 169 case histories from a specialized infectious psychiatric department (May 2020 to January 2021) were evaluated. Progression indicators of severe and mild COVID-19, along with the duration of persistent SARS-CoV-2 viral shedding, were assessed in correlation with the administration of antidepressants, antipsychotics, and acid sphingomyelinase inhibitors (FIASMA-active drugs).

**Results:**

The use of any psychotropic agents was associated with a 0.9% increase in the risk of severe course of COVID-19 for each unit increase in the systemic inflammation index PLR, specifically in patients with intellectual disability (ICD-10 codes F70-79), when compared to patients with schizophrenia (ICD-10 codes F20-29): R²McF=0.138; AIC=181; χ²=25.8; df=9; p=0.002. High PLR values and the use of FIASMA-active drugs were associated with prolonged COVID-19 duration, while antidepressant therapy and elevated C-reactive protein levels were associated with a reduced predicted duration of viral shedding in 13.8% of variance: R²=0.0864; AIC=1299; F=5.2(3), p=0.002. Including the nosology of psychiatric disorders in the regression model increased the proportion of explained variance to 22.8%.

**Conclusions:**

Thymoanaleptic therapy for individuals with psychiatric disorders may act as a protective factor against COVID-19. There is no evidence suggesting adverse effects of antipsychotics on the severity and duration of COVID-19. Further research is necessary to investigate the effects of FIASMA-active psychopharmacological agents within nosologically homogeneous groups.

**Disclosure of Interest:**

None Declared

